# Toxicity analysis of food contaminants based on network toxicology and molecular docking: A case study of Zearalenone

**DOI:** 10.1371/journal.pone.0349937

**Published:** 2026-06-26

**Authors:** Xiaobo Xu, RuiLi Miao, Ping Xu, Leishan Chen, Kun Zhao, PingHui Xu, Hanna Fotina

**Affiliations:** 1 School of Biological Engineering, Xinxiang University, Xinxiang, China; 2 College of Animal Science and Veterinary Medicine, Henan Institute of Science and Technology, Xinxiang, China; 3 Xinxiang Bokai Biotechnology Co., Ltd, Xinxiang, China; 4 Laboratory of Molecular Biology of Ticks, Department of Disease Vectors, Institute of Parasitology, Biology Centre CAS, Branisovska, Ceske Budejovice, Czech Republic; USDA-ARS Southeast Area, UNITED STATES OF AMERICA

## Abstract

Zearalenone (ZEA) is a non – steroid estrogen contaminant produced by *fungi* of the genus Fusarium. It is widely present in grains and seriously threatens global food safety. This study takes ZEA as an example to effectively evaluate the toxicity of food contaminants through network toxicology, molecular docking and molecular dynamics simulation strategies, and analyzes the molecular mechanism of its hypothesized toxicity – nephrotoxicity. The study found that ZEA affects immunotoxicity, inflammatory response, apoptosis, and cytogenetic mutations by regulating core targets such as STAT3, ESR1, HSP90AA1, MMP9, and MTOR, and regulating pathways such as the cancer pathway and the PI3K - Akt signaling pathway, ultimately leading to nephrotoxicity. The results of molecular docking and molecular dynamics simulation also showed good binding affinity between ZEA and core targets. This result provided a theoretical basis for understanding the molecular mechanism of ZEA – nephrotoxicity and for preventing and treating cancers caused by the food contaminant ZEA. In addition, the methods of network toxicology and molecular docking also provided an effective way to rapidly evaluate the toxicity of food contaminants, effectively addressing the cost and ethical issues associated with the use of experimental animals.

## Introduction

Against the backdrop of the rapid development of the global food supply chain and food processing technology, the toxicity assessment of food contaminants has become one of the core issues in ensuring public health [1]. Mycotoxins, as one of the major contaminants, pose a severe challenge to food safety due to their strong concealment, persistent toxicity, and easy accumulation in grains [2]. Among them, zearalenone (ZEA) is metabolically produced by fungi of the genus *Fusarium spp* under specific temperature and humidity conditions. It widely contaminates crops such as corn and wheat [3]. Due to its chemical structure being highly similar to endogenous estrogens, ZEA can trigger multiple biological toxic effects by mimicking or antagonizing the functions of estrogen receptors [4–5]. Numerous studies have shown that ZEA can not only cause reproductive dysfunction, immunosuppression, and endocrine disorders but also is closely related to systemic pathological changes such as liver and kidney injuries [6–7]. For example, ZEA can interfere with the activities of multiple enzymes in the liver, thereby affecting the normal metabolism of the liver [8]. However, at present, the multi – target action mechanism of ZEA has not been fully elucidated, and there are still many unknown areas that need to be explored. Therefore, a comprehensive and systematic analysis of the toxicity network of ZEA and its molecular regulatory mechanism is of great significance for formulating precise and effective prevention and control strategies and reducing the risk of food – borne diseases.

Traditional toxicology methods (such as animal experiments) can provide intuitive toxicity data, but their time – consuming nature, high cost, and ethical controversies limit their application in rapid screening. In addition, the single – target research paradigm is difficult to fully reveal the complex toxic effects caused by contaminants such as ZEA through the coordination of multiple pathways [9]. In recent years, the cross – integration of bio-informatics and computational toxicology has provided innovative tools for toxicity assessment [10]. Network toxicology can construct an interactive network of contaminants – targets – pathways by integrating multi – source data such as genomics, proteomics, and metabolomics, and systematically identify the core toxic targets and key signaling pathways. Molecular docking method can generate the binding conformation of small – molecule ligands to target proteins, quantitatively predict their binding affinity and interaction patterns, and provide visual evidence at the molecular level for mechanism research [11]. The combination of the two not only breaks through the limitations of traditional methods but also provides an efficient and low – cost research framework for analyzing the multi – dimensional toxic mechanisms of contaminants.

Although previous studies have preliminarily revealed the estrogen – like effect and reproductive toxicity of ZEA [12], there are still significant research gaps in its injury mechanism to non – reproductive organs (such as the kidneys). Existing research results mostly focus on single targets or pathways and fail to integrate the systemic toxicity network caused by ZEA through pathways such as inflammatory response, apoptosis, and metabolic regulation.

In this study, we analyzed the potential nephrotoxicity mechanism of ZEA by network toxicology. Then, molecular docking and molecular dynamics simulation analyses were used to estimate the binding stability. The results of this study not only provide a new paradigm for the toxicity assessment of ZEA, but also lay a theoretical foundation for the rapid screening of food contaminants and precise risk management.

## Methods

### Preliminary network analysis of ZEA toxicity

The SDF file of ZEA was obtained from PubChem database (https://pubchem.ncbi.nlm.nih.gov/), and then the Structural information of ZEA was imported into ProTox – 3.0 (https://tox.charite.de/protox3/index.php?site) and ADMETlab2.0 (https://admetmesh.scbdd.com/) to make a preliminary prediction of the toxicity of ZEA in accordance with the integration of the network search algorithm. ProTox – 3.0 uses a combination of machine learning algorithms and chemical structure – activity relationships to predict toxicity endpoints, while ADMETlab2.0 predicts ADMET (absorption, distribution, metabolism, excretion, and toxicity) properties through a series of computer – simulated models. The integration of these two platforms provides a more comprehensive prediction of ZEA toxicity.

### Target collection of ZEA

After inputting Zearalenone into the PubChem database to obtain its SMILES number, the SMILES number was then inputted into the TargetNet (http://targetnet.scbdd.com/) database, the SEA database (with non-human proteins removed), and the Swiss Target Prediction (http://swisstargetprediction.ch/) database (>0.6) to screen for the targets of ZEA, and the species was limited to “*Homo sapiens*.” Subsequently, the potential targets of ZEA were retrieved. The search results were then combined and duplicates were removed, and the Uniprot database (https://www.uniprot.org/) was used to standardize the obtained target names. Each database has its unique algorithm for predicting targets. TargetNet uses a network – based method, while Swiss Target Prediction is based on ligand – similarity search. The combination of these two databases can improve the comprehensiveness of target prediction.

### Screening of nephrotoxicity related targets

GeneCards (https://www.genecards.org/) (Relevance score>20) and CTD (https://ctdbase.org/) databases (Inference Score>130) was utilized to verify the disease as “kidney injury,” “nephrotoxicity,” and “kidney dysfunction.” After merging and removing duplicate targets, the intersected targets in the Venn plot were defined as potential targets of ZEA associated with nephrotoxicity.

### Protein - protein interaction analysis and core target screening

A protein-protein interaction (PPI) network was generated to identify interacting proteins. The intersecting genes of ZEA potential nephrotoxicity targets were entered into the STRING (http://string-db.org, Version 12.0) database, with the species limited to “*Homo sapiens*” and interaction score >0.4. The Cytoscape (https://cytoscape.org/, v.3.7.2) plug-ins Cytohubba was used to analyze the topological parameters and select the core targets. The top 5 core targets were selected for molecular docking analysis.

### Enrichment analysis of target protein gene functions and pathways

Gene Ontology (GO) enables the analysis of gene function based on the biological processes (BP), cellular components (CC), and molecular functions (MF). Kyoto Encyclopedia of Genes and Genomes (KEGG) pathway enrichment analysis enables an understanding of the biological pathways associated with genes. The obtained intersection targets were inputted in the Metascape database (https://www.metascape.org/), and the cut-off *p*-value, min overlap and enrichment value were set to 0.01, 3 and 1.5. According to the *p*-value, the bioinformatics platform (https://www.bioinformatics.com.cn) was used to visualize the top 10 GO enrichments and top 10 KEGG pathways results as bar and bubble plots. Finally, the complex regulatory network of ZEA associated with nephrotoxicity was shown by the “target-pathway-disease” network, incorporating the elements of ZEA, potential target, and top 10 enriched pathway, and visualized by Cytoscape software (https://cytoscape.org/, v.3.7.2).

### Molecular docking of ZEA and core targets

The protein crystal structures used for docking were obtained from the Protein Data Bank (PDB). The PDB IDs for HSP90AA1, MMP9, mTOR, and STAT3 were 5T01, 6IQ6, 6QHD, and 6NJS, respectively. The 3D structure of the small molecule Zearalenone (Compound CID: 5281576) was downloaded from the PubChem database and energy-minimized using the MMFF94 force field.

Molecular docking was performed using AutoDock Vina 1.2.7 software [13]. Prior to docking, the receptor proteins were processed with PyMol 2.5.2 [14], which included the removal of water molecules, salt ions, and other small molecules. A docking box was then defined to encompass the crystallographic ligand. Subsequently, all processed small molecules and receptor proteins were converted into the PDBQT format required by AutoDock Vina 1.2.7 using ADFRsuite 1.0 [15]. During docking, the exhaustiveness of the global search was set to 32, while all other parameters remained at their default settings. The output docking conformation with the highest score was considered the binding conformation. Finally, PyMol 2.5.2 was used for the visualization and analysis of the docking results.

### Molecular dynamics simulation verified the binding stability

The complex of the small molecule and protein obtained through docking with AMBER 24 software was used as the initial structure for all-atom molecular dynamics (MD) simulations to further investigate the stability of the small-molecule ligand within the protein [16]. Prior to simulation, the AM1-BCC charges for the small molecule were calculated using the antechamber module [17]. Subsequently, the small molecule and the protein were described using the GAFF2 small molecule force field and the ff14SB protein force field, respectively [18,19]. For each system, hydrogen atoms were added using the LEaP module. A truncated octahedral TIP3P solvent box was added at a distance of 10 Å from the system, and Na^+^/Cl^-^ ions were added to neutralize the system charge [20]. Finally, the topology and parameter files for the simulation were generated.

Concurrently, energy minimization of the system was conducted, comprising 2500 steps of steepest descent followed by 2500 steps of conjugate gradient minimization. Following energy minimization, the system was gradually heated from 0 K to 298.15 K over 200 ps in the NVT (canonical) ensemble with a constant volume and a controlled heating rate. Subsequently, a 500 ps NVT ensemble simulation was performed at a constant temperature of 298.15 K to allow for further equilibration of the solvent distribution within the box. Finally, the system was equilibrated for 500 ps under NPT (isothermal-isobaric) conditions. The complex system was then subjected to a 100 ns production simulation under NPT ensemble conditions with periodic boundary conditions applied. During the simulations, a non-bonded cutoff distance of 10 Å was used. Long-range electrostatic interactions were calculated using the Particle Mesh Ewald (PME) method [21]. Bond lengths involving hydrogen atoms were constrained using the SHAKE algorithm [22]. Temperature control was maintained using the Langevin thermostat with a collision frequency (γ) of 2 ps ⁻ ¹ [23]. The system pressure was maintained at 1 atm. The integration time step was set to 2 fs, and trajectory frames were saved every 10 ps for subsequent analysis.

Regarding trajectory analysis, the 100 ns simulation trajectory was processed using the cpptraj module. The root-mean-square deviation (RMSD) was calculated based on non-hydrogen atoms of the small molecule and the protein. The root-mean-square fluctuation (RMSF) was calculated using the Cα atoms of the protein backbone. The radius of gyration (Rg) was calculated based on the non-hydrogen atoms of the small molecule and the protein.

### MM/PBSA analysis

The binding free energy between the protein and ligand in all systems was calculated using the MM/PBSA method [24–26]. In this study, MD trajectories from 90–100 ns were employed for the calculations, with the specific formula as follows:


$$\begin{array}{c} {\Delta G}_{bind}={\Delta G}_{\text{complex}}\;-\;({\Delta G}_{\text{receptor}}+\;{\Delta G}_{\text{ligand}})\;={\;\Delta E}_{\text{internal}}+{\Delta E}_{\text{VDW}}+{\Delta E}_{\text{elec}}{+\Delta G}_{\text{PB}}+{\Delta G}_{\text{SA}} \end{array}$$ (1)

In Equation (1), ${\Delta \text{E}}_{\text{internal}}$ represents the internal energy, ${\Delta \text{E}}_{\text{VDW}}$ denotes the van der Waals interaction, and ${\Delta \text{E}}_{\text{elec}}$ represents the electrostatic interaction. The internal energy includes bond energy (Ebond), angle energy (Eangle), and torsional energy (Etorsion). ${\Delta \text{G}}_{\text{PB}}$ and ${\Delta \text{G}}_{\text{SA}}$are collectively referred to as the solvation free energy, where G_PB_ is the polar solvation free energy and G_SA_ is the nonpolar solvation free energy. For ${\Delta \text{G}}_{\text{PB}}$, the PB8 model was used for calculation. The nonpolar solvation free energy (ΔG_SA_) was calculated based on the product of the surface tension (γ) and the solvent-accessible surface area (SA), using the formula ΔG_SA_ = 0.0072 × ΔSASA [27]. Entropy changes were not considered in this study due to their high computational cost and low accuracy [24].

## Results

### Preliminary Network Analysis of ZEA Toxicity

**Fig 1:**
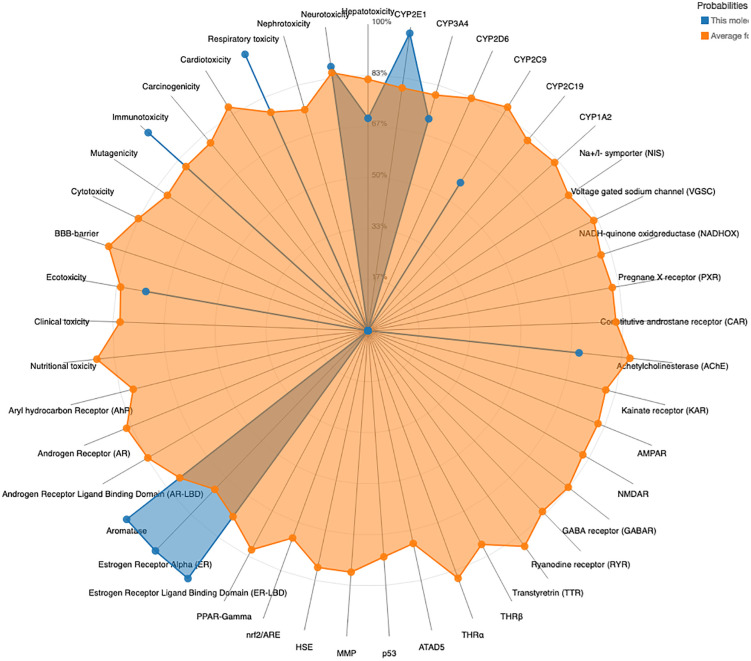
Toxicity analysis results of ZEA.

By integrating the toxicity analysis results of ZEA from ProTox – 3.0 and ADMETlab2.0, we obtained a basic summary of ZEA toxicity (Fig 1). The results showed that the active toxicity endpoints were closely related to nephrotoxicity. Through research, it has been found that ZEA binds to estrogen receptors in kidney cell proteins, interfering with cellular metabolism [28]. These findings were consistent with previous studies, which verified the damage of ZEA to kidney function through cell experiments and animal models [29]. This study not only verified the previous results but also provided a basis for in – depth research on ZEA toxicity. Follow – up in – vitro and in – vivo experiments with different doses and exposure times can be carried out to provide a basis for food safety standards and prevention and control strategies.

### Identification of ZEA Associated with Nephrotoxicity Targets

**Fig 2:**
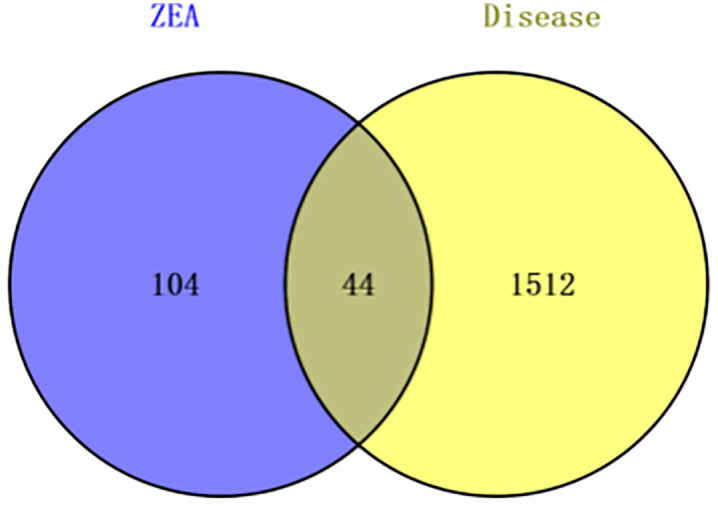
Veen diagram of potential targets to the ZEA associated with nephrotoxicity.

In this study, 148 ZEA targets were screened from the TargetNet database, SEA database, and SwissTargetPrediction database. Besides, 1556 targets highly related to nephrotoxicity were retrieved from the GeneCards and CTD databases. 44 targets overlapped between toxic ZEA and nephrotoxicity were obtained by Venn (Fig 2).

### Interaction Network of Potential Targets and Acquisition of Core Genes

**Fig 3:**
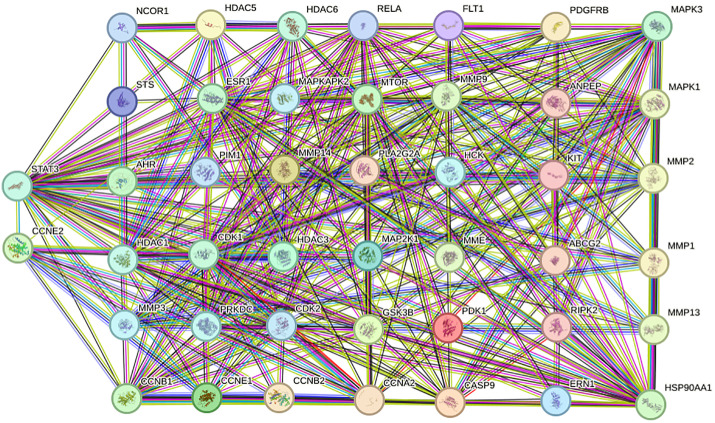
The 44 targets PPI network obtained from STRING database.

**Fig 4:**
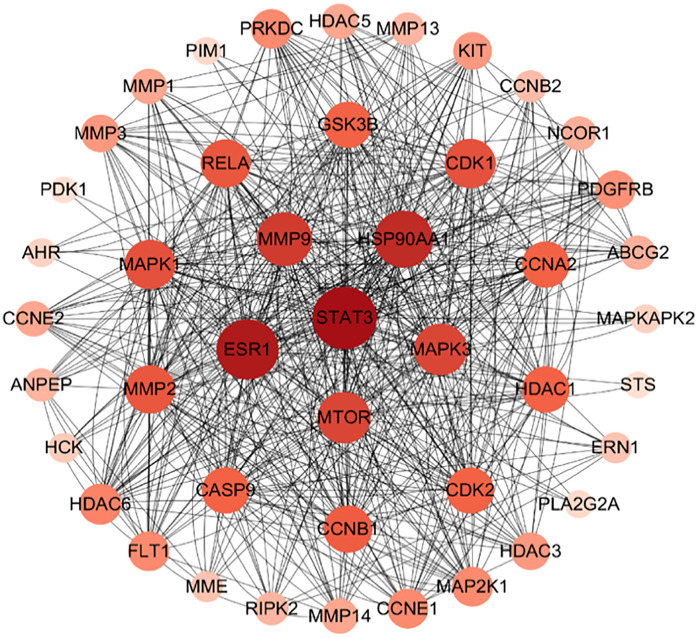
Core targets of the ZEA associated with nephrotoxicity.

The 44 intersection targets were incorporated into the STRING database to construct the PPI network. 44 nodes and 576 edges of protein-protein interaction were visually displayed in Cytoscape software, topological analysis including degree centrality (DC) and combined score, and the maximal clique centrality (MCC) algorithm was further used to screen the core targets. The top 5 targets as the core targets in descending order were STAT3, ESR1, HSP90AA1, MMP9, and MTOR (Fig 3, Fig 4).

### GO Function Enrichment Analysis and KEGG Pathway Enrichment Analysis

**Fig 5:**
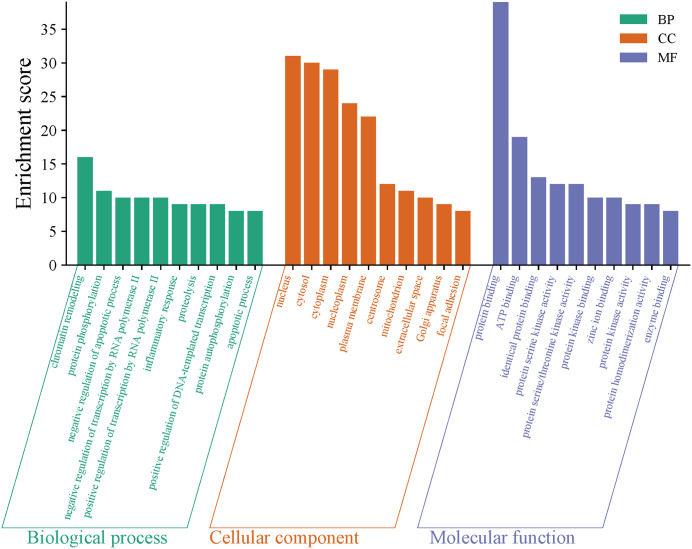
The bar graph of top 10 BP, CC and MF items in GO functional enrichment analysis diagram of the targets related to ZEA associated with nephrotoxicity.

**Fig 6:**
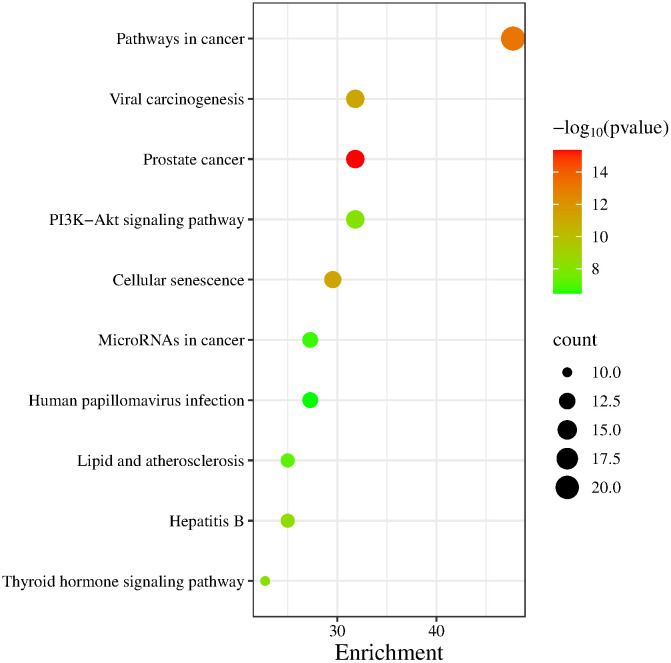
The bubble diagram of the top 10 KEGG functional enrichment analysis of the targets related to ZEA associated with nephrotoxicity.

The analysis generated 355 statistically significant GO entries, including 219 biological processes (BP), 31 cellular components (CC), and 105 molecular functions (MF). And 117 enriched signaling pathways were visually represented. The top 10 biological processes included the chromatin remodeling, protein phosphorylation, negative regulation of apoptotic process, negative regulation of transcription by RNA polymerase II, positive regulation of transcription by RNA polymerase II, inflammatory response, proteolysis, positive regulation of DNA-templated transcription, protein autophosphorylation and apoptotic process (Fig 5). The top 10 significantly enriched KEGG pathways included Pathways in cancer, Viral carcinogenesis, Prostate cancer, PI3K-Akt signaling pathway, Cellular senescence, MicroRNAs in cancer, Human papillomavirus infection, Lipid and atherosclerosis, Hepatitis B and Thyroid hormone signaling pathway (Fig 6).

**Fig 7:**
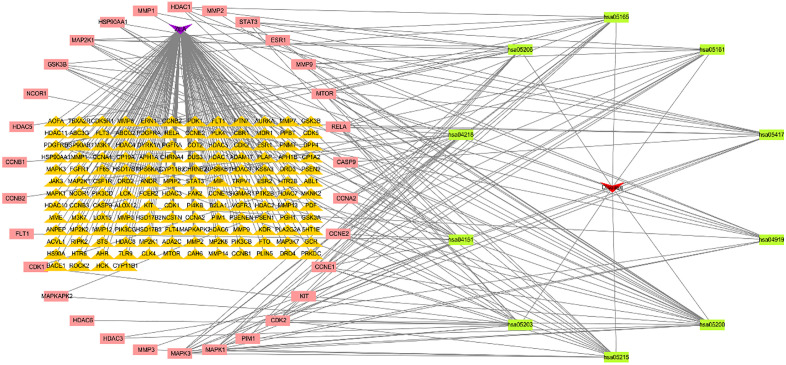
The Target – Pathway – Disease network map showed the complex regulation of ZEA associated with nephrotoxicity.

After identifying the core targets and the top 10 pathways from previous analysis, a “target-pathway-disease” network was constructed by Cytoscape to reveal the complex molecular mechanism of ZEA associated with nephrotoxicity (Fig 7).

### Molecular docking of ZEA and core target proteins in nephrotoxicity

Molecular docking simulation is a convenient and effective method for investigating the interactions between small molecules and target proteins. Here, we employed AutoDock Vina 1.2.7 software to perform docking studies between the compounds and the proteins. By analyzing the relative position of the ligand ZEA within the binding pocket of each target protein and its interaction with the receptor amino acid residues, as shown in the figures, distinct differences in the amino acid composition and spatial distribution of the ZEA binding sites among the different target proteins could be clearly distinguished.

**Fig 8:**
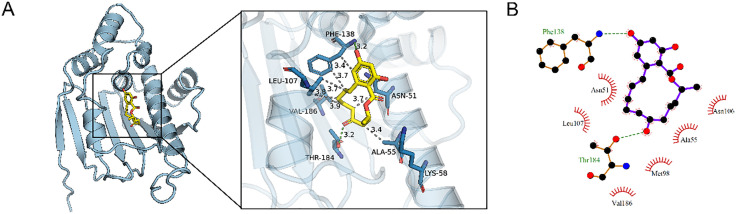
The binding mode of HSP90AA1_ZEA obtained through molecular docking. **A)** The left panel shows the overall view, and the right panel shows a detailed view. In the figure, the small molecule is represented as a yellow stick model, the protein as a cyan cartoon model, green dashed lines indicate hydrogen bond interactions, and gray dashed lines indicate hydrophobic interactions. **B)** A 2D interaction diagram.

The docking results indicated that in the HSP90AA1/ZEA complex (Fig 8), Zearalenone was primarily embedded within the hydrophobic pocket of HSP90AA1. Tight hydrophobic packing was formed with residues such as ASN-51, ALA-55, PHE-138, VAL-186, and LEU-107. Notably, the aromatic side chain of PHE-138 provided crucial hydrophobic complementarity within the pocket. In addition to hydrophobic interactions, stable hydrogen bonds were formed between Zearalenone and PHE-138 as well as THR-184, further locking its orientation and resulting in a relatively fixed conformation within the binding cavity. These interactions collectively ensured the binding stability and positional specificity of the ligand.

**Fig 9:**
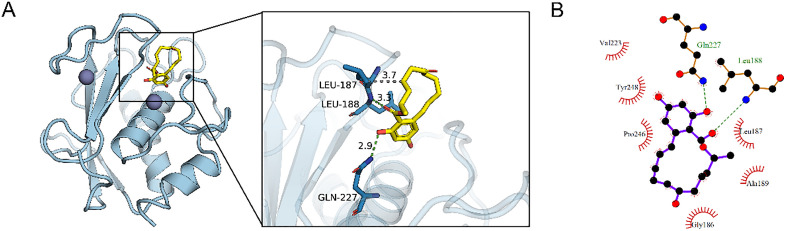
The binding mode of MMP9_ZEA obtained through molecular docking. **A)** The left panel shows the overall view, and the right panel shows a detailed view. In the figure, the small molecule is represented as a yellow stick model, the protein as a cyan cartoon model, green dashed lines indicate hydrogen bond interactions, and gray dashed lines indicate hydrophobic interactions. **B)** A 2D interaction diagram.

In the MMP9/ZEA complex (Fig 9), the ligand primarily accessed the binding cavity of MMP9 via hydrophobic contact with LEU-187, while simultaneously forming hydrogen bonds with LEU-188 and GLN-227. This combined hydrophobic-hydrogen bond interaction pattern enabled stable anchoring of the ligand at the active site of MMP9. The polar side chain of GLN-227 likely played a significant role in modulating the spatial positioning of the ligand, thereby supporting a relatively stable binding state.

**Fig 10:**
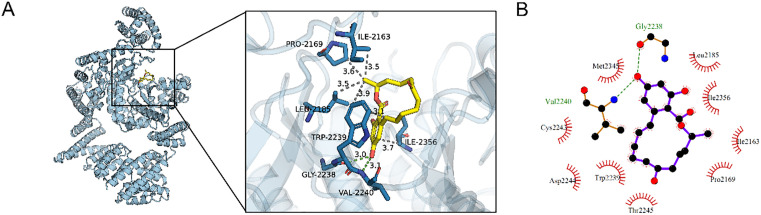
The binding mode of mTOR_ZEA obtained through molecular docking. **A)** The left panel shows the overall view, and the right panel shows a detailed view. In the figure, the small molecule is represented as a yellow stick model, the protein as a cyan cartoon model, green dashed lines indicate hydrogen bond interactions, and gray dashed lines indicate hydrophobic interactions. **B)** A 2D interaction diagram.

The mTOR/ZEA complex (Fig 10) exhibited typical deep hydrophobic pocket binding characteristics. Zearalenone formed tight hydrophobic associations with numerous hydrophobic residues, including ILE-2163, ILE-2356, PRO-2169, LEU-2185, and TRP-2239. The large side chain of TRP-2239 provided additional hydrophobic support for the ligand. Furthermore, a hydrogen bond network involving VAL-2240 and GLY-2238 enhanced the positioning accuracy of the ligand, maintaining its stable posture within the mTOR binding site. This synergistic hydrophobic-hydrogen bond interaction is a typical feature of ligand binding to mTOR.

**Fig 11:**
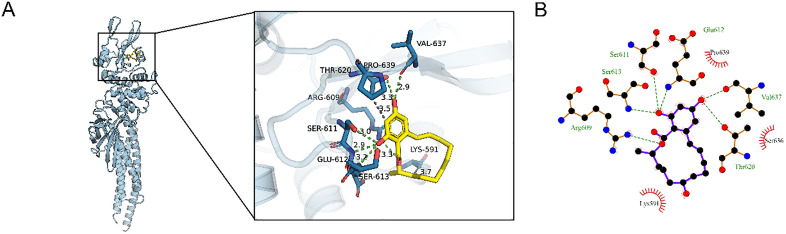
The binding mode of STAT3_ZEA obtained through molecular docking. **A)** The left panel shows the overall view, and the right panel shows a detailed view. In the figure, the small molecule is represented as a yellow stick model, the protein as a cyan cartoon model, green dashed lines indicate hydrogen bond interactions, and gray dashed lines indicate hydrophobic interactions. **B)** A 2D interaction diagram.

In the STAT3/ZEA complex (Fig 11), the ligand was embedded within a hydrophobic-semi-polar pocket formed jointly by LYS-591 and PRO-639, establishing initial hydrophobic stability through these residues. Concurrently, a multiple hydrogen bond network was formed between Zearalenone and SER-611, GLU-612, SER-613, VAL-637, ARG-609, and THR-620. These extensive polar interactions significantly increased the binding robustness of the ligand and helped maintain its highly specific anchored position within the active conformation of STAT3, reflecting the dominant contribution of hydrogen bonds to the binding mode in this system.

The docking scores for each molecule and protein were shown in Table 1. Typically, a lower value is considered to indicate a greater likelihood of binding.

**Table 1:** The docking scores for each molecule and protein.

Target_name	Ligand_name	Docking_score(kcal/mol)
HSP90AA1	Zearalenone	−7.891
MMP9	Zearalenone	−7.319
STAT3	Zearalenone	−6.16
mTOR	Zearalenone	−7.539

### Molecular dynamics simulation

**Fig 12:**
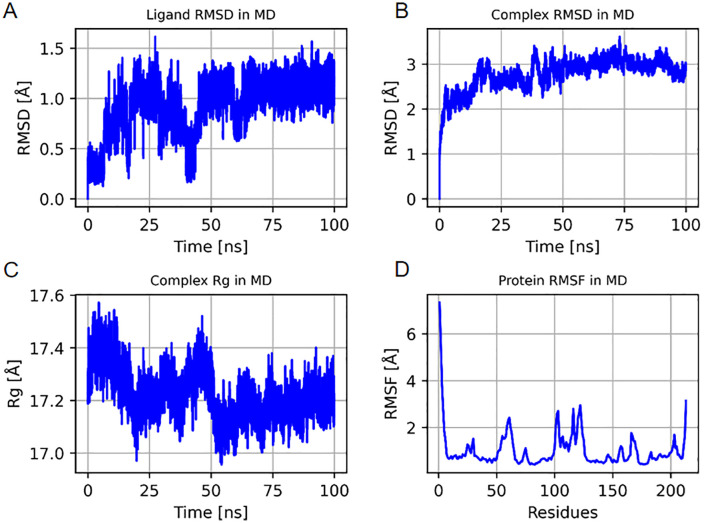
Molecular dynamic simulations results of HSP90AA1-ZEA. **(A)** Variation of ligand RMSD (Ligand RMSD in MD), **(B)** complex RMSD (Complex RMSD in MD), and **(C)** complex radius of gyration Rg (Complex Rg in MD) with simulation time during the simulation. **(D)** Protein RMSF (Protein RMSF in MD).

Molecular dynamics simulation is an important technique for analyzing the conformational changes and stability of ligand-protein complexes after docking. To further explore the binding stability of ZEA with HSP90AA1 (−7.891 kcal/mol), the RMSD, Rg, and RMSF curves were calculated. The RMSD curve represents the fluctuation of the protein conformation.The RMSD of the ligand ZEA in the binding pocket of HSP90AA1 (Fig 12A) showed a trend of initial gradual increase followed by oscillation within a relatively stable range over time. In the initial stage (0–10 ns), the RMSD was low, then gradually increased and subsequently maintained stable oscillations within approximately 0.8–1.5 Å for the remaining ~70 ns. This change suggests that the ligand underwent necessary conformational adaptation in the early stage to match the binding cavity of HSP90AA1, followed by entry into a relatively stable binding mode. The overall RMSD magnitude fell within the acceptable range typical for stably bound ligands, indicating that the interaction between the ligand and the protein remained stable throughout the 100 ns simulation, with no signs of significant dissociation or escape.

The RMSD of the HSP90AA1/ZEA complex (Fig 12B) increased rapidly at the beginning of the simulation, reached a plateau around 20 ns, and subsequently maintained minor fluctuations within the range of 2.5–3.1 Å. This RMSD behavior indicates that the main protein structure completed conformational relaxation and energy equilibration in the initial stage, after which the overall conformation remained stable, with no significant structural collapse or large-scale deformation. Considering the trend of the ligand RMSD, the complex overall exhibited a stable binding conformation, suggesting that small molecule binding did not cause structural disruption or abnormal perturbation of HSP90AA1.

The radius of gyration (Rg) curve represents the compactness of the overall protein structure. The Rg of the complex gradually decreased with simulation time and entered a convergence interval around 50 ns, after which it fluctuated within approximately 17.0–17.4 Å (Fig 12C). The radius of gyration reflects the compactness of the overall protein conformation. The stable plateau of Rg here indicates that HSP90AA1 tended to form a stable structural compactness after binding Zearalenone, with neither significant expansion nor obvious structural contraction. The minor fluctuations in Rg can be regarded as a normal phenomenon during the natural breathing-like conformational changes of the protein, and the overall stable plateau further supports the maintenance of structural integrity of the complex during the simulation.

The RMSF curve represents the fluctuation of amino acid residues in the protein. The RMSF distribution of the protein showed low flexibility (approximately 1–2 Å) in most regions, indicating a stable main structure (Fig 12D). RMSF peaks were mainly concentrated in the N-terminus, C-terminus, and several loop segments, which correspond to the inherently high-flexibility disordered regions in the protein structure. No abnormally high fluctuations were observed in key functional domains or the ligand-binding region, indicating that the binding of Zearalenone did not induce local structural instability. The binding pocket remained in a steady-state conformation, which is conducive to maintaining effective drug recognition and binding.

### MM/PBSA results

Based on the trajectories from molecular dynamics simulations, the binding free energy was calculated using the MM-PBSA method, which can more accurately reflect the binding effect between the small molecule and the target protein. As shown in the Table 2, the binding free energy of HSP90AA1/Zearalenone is −13.21 ± 1.66 kcal/mol. A negative value indicates that the molecule exhibits binding affinity toward the target protein, with lower values corresponding to stronger binding. Our calculations clearly demonstrate that the HSP90AA1/Zearalenone complex possesses binding potential. Through energy decomposition, it can be observed that the main contributions to their binding come from electrostatic energy and van der Waals energy, followed by non‑polar solvation free energy.

**Table 2:** Binding free energies and energy components predicted by MM/PBSA (kcal/mol).

System name	HSP90AA1/Zearalenone
ΔE_vdw_ (van der Waals energy)	−42.72 ± 1.03
ΔE_elec_ (electrostatic energy)	−20.30 ± 4.58
ΔG_PB_ (electrostatic contribution to solvation)	55.36 ± 3.65
ΔG_SA_ (non-polar contribution to solvation)	−5.55 ± 0.23
ΔG_bind_ (binding free energy)	−13.21 ± 1.66

## Discussion

This study systematically analyzed the potential toxicity and molecular mechanism of zearalenone (ZEA) by means of network toxicology and molecular docking techniques, and obtained a series of valuable results. The research findings clearly demonstrate that ZEA has extensive and complex toxic effects, among which the damage to the kidneys is particularly notable, as it can lead to kidney injury and trigger kidney dysfunction diseases.

Regarding kidney injury, the toxicity network constructed by network toxicology technology clearly shows multiple potential pathways through which ZEA exerts toxic effects on the kidneys. Different from traditional single – factor studies on kidney injury, network toxicology integrates multi – omics data and comprehensively reveals the molecular mechanism by which ZEA interferes with the normal physiological functions of the kidneys. For example, ZEA induces degeneration and swelling of renal proximal tubules, leading to lumen narrowing, as well as mitochondrial damage and autophagosome formation in cells, indicating that it may affect the activity of enzymes involved in renal energy metabolism by disrupting mitochondrial function [15]. These enzymes play a crucial role in maintaining the normal energy supply and material metabolism balance of kidney cells. ZEA’s interference with them can lead to insufficient energy supply in estrogen receptors in kidney cell proteins, thereby triggering cell damage. At the same time, ZEA also affects the redox balance in the kidneys. By upregulating the level of reactive oxygen species (ROS) in kidney tissues, it activates oxidative stress – related signaling pathways [30–32]. When oxidative stress is imbalanced, excessive ROS will attack the biological membranes, proteins, and nucleic acids of kidney cells, resulting in damage to cell structure and function. This is one of the important mechanisms by which ZEA causes kidney injury. Previous studies [33] have also pointed out that ZEA exposure can cause changes in oxidative stress indicators in kidney tissues, which is consistent with the results of this study and further confirms this mechanism.

Regarding kidney dysfunction diseases, long – term exposure to ZEA can cause significant changes in kidney function indicators. Traditional research, when analyzing kidney dysfunction diseases, often focuses on the detection of several common clinical indicators and has difficulty delving into the molecular mechanism of its pathogenesis. In contrast, this study uses network toxicology and molecular docking techniques to deeply analyze how ZEA affects kidney function through multi – target and multi – pathway effects. For example, ZEA can interfere with the filtration function of the kidneys, leading to an increase in kidney function indicators such as serum creatinine and blood urea nitrogen [34,35]. At the molecular mechanism level, ZEA affects the expression of proteins related to glomerular podocytes, such as nephrin and podocin, disrupting the integrity of the glomerular filtration barrier and causing the leakage of macromolecules such as proteins, thus affecting the normal filtration function of the kidneys [36,37]. In addition, ZEA also affects the reabsorption and secretion functions of the renal tubules. By interfering with the ion transport proteins of renal tubular epithelial cells, such as sodium – potassium ATPase and bicarbonate transporters, it causes disorders in the body’s electrolyte balance and further aggravates kidney dysfunction [38,39]. Recent studies have also shown that ZEA exposure can lead to changes in the expression of proteins related to glomerular and tubular functions, which is consistent with the conclusions of this study [40].

From the results of molecular docking, ZEA has a strong binding effect with multiple key target proteins in the kidneys. The complex structure formed by ZEA and the protein HSP90AA1 is the most stable, with a binding energy of −7.891 kcal/mol, indicating that there is a strong affinity between them. HSP90AA1 is an important member of the heat shock protein 90 (Hsp90) family, which functions to maintain protein homeostasis and assist in the folding and activation of other proteins [41], the interactions of ZEA with it may play an indispensable role in the toxic mechanism of ZEA. Further molecular dynamics simulation analysis was performed on the HSP90AA1/ZEA complex. The results indicated that the interaction between the ligand and the protein remained stable throughout the entire 100 ns simulation, with no significant dissociation or escape observed. The overall complex exhibited a stable binding conformation, indicating that the binding of the small molecule did not cause structural disruption or abnormal perturbation of HSP90AA1. Moreover, after binding ZEA, HSP90AA1 tended to form a stable structural compactness, with neither significant expansion nor obvious structural contraction observed.

This molecular docking and molecular dynamics simulation study of ZEA and key target proteins has more clearly defined the potential molecular mechanism by which ZEA exerts its toxic effects, laying a foundation for further in – depth research on ZEA toxic effects and the formulation of targeted prevention and control strategies. Follow – up research can focus on the specific regulatory mechanisms of relevant signaling pathways after the interaction between ZEA and HSP90AA1, and verify them at the cell and animal levels. Multi – omics methods such as gene – edited mouse models, cell imaging technology, metabolomics, and proteomics analysis can be used to comprehensively reveal the toxic mystery of ZEA.

In summary, this in – depth study of ZEA toxicity through network toxicology and molecular docking techniques provides key evidence for a comprehensive understanding of the impact of ZEA on kidney injury and kidney dysfunction diseases, and also fully demonstrates the important value of these two emerging techniques in evaluating the kidney toxicity of food contaminants.

## Supporting information

S1 File Raw data. (ZIP)
